# Development of a species-specific transformation system using the novel endogenous promoter calreticulin from oleaginous microalgae *Ettlia* sp.

**DOI:** 10.1038/s41598-020-70503-2

**Published:** 2020-08-18

**Authors:** Jun-Woo Lee, Min-Woo Lee, Ji-San Ha, Dae-Soo Kim, EonSeon Jin, Hyung-Gwan Lee, Hee-Mock Oh

**Affiliations:** 1 Cell Factory Research Center, Korea Research Institute of Bioscience and Biotechnology (KRIBB), Daejeon, Republic of Korea; 2 Department of Life Science, Hanyang University, Seoul, Republic of Korea; 3 Department of Environmental Biotechnology, University of Science and Technology (UST), Daejeon, Republic of Korea; 4 Department of Biological Sciences, Sungkyunkwan University, Suwon, Republic of Korea; 5 Rare Disease Research Center, Korea Research Institute of Bioscience and Biotechnology (KRIBB), Daejeon, Republic of Korea

**Keywords:** Biological techniques, Biotechnology

## Abstract

Microalgae not only serve as raw materials for biofuel but also have uses in the food, pharmaceutical, and cosmetic industries. However, regulated gene expression in microalgae has only been achieved in a few strains due to the lack of genome information and unstable transformation. This study developed a species-specific transformation system for an oleaginous microalga, *Ettlia* sp. YC001, using electroporation. The electroporation was optimized using three parameters (waveform, field strength, and number of pulses), and the final selection was a 5 kV cm^−1^ field strength using an exponential decay wave with one pulse. A new strong endogenous promoter CRT (Pcrt) was identified using transcriptome and quantitative PCR analysis of highly expressed genes during the late exponential growth phase. The activities of this promoter were characterized using a codon optimized cyan fluorescent protein (CFP) as a reporter. The expression of CFP was similar under Pcrt and under the constitutive promoter psaD (PpsaD). The developed transformation system using electroporation with the endogenous promoter is simple to prepare, is easy to operate with high repetition, and utilizes a species-specific vector for high expression. This system could be used not only in molecular studies on microalgae but also in various industrial applications of microalgae.

## Introduction

The last decade of microalgae research has greatly expanded both fundamental knowledge and industrial applications, which include the biofuel, cosmetics, pharmaceutical and nutraceutical industries^1^. Strain development is an essential prerequisite to create even more diverse biotechnological applications and to enable microalgal commercialization to generate desirable industrial strains that can produce various types of valuable compounds by using forward and reverse genetic methods^2^. Chemical treatments and radiation exposure are suitable methods for random mutagenesis^3^ but are time consuming and labour intensive, and the transformed characteristics could easily be reversed due to DNA repair and the unstable maintenance of modified genes^4^. However, the metabolic engineering of microalgae offers an alternative method for increasing metabolites without impairing growth by regulating only the gene expression related to the targeted metabolic pathway^5–7^. However, the genetic modification of microalgae requires not only genome information to identify target genes that regulate biosynthesis pathways and screening expression elements but also the development of species-specific delivery methods and gene expression systems^8,9^.

*Ettlia* sp. YC001 (hereinafter referred to as *Ettlia* sp.), isolated from Daejeon, Korea, is an indigenous microalga with high-value industrial properties. It can tolerate up to 10% CO_2_, and the total lipid accumulation can reach up to 55% under depleted nitrogen conditions^10^. The high growth rate and high adaptation, regardless of whether cultivation indoors and/or outdoors, were characterized from a lab-scale culture to an open raceway pond. The optimized culture conditions for *Ettlia* sp. have also been determined to yield a maximized biomass productivity of 1.67 g L^−1^ d^−1^ and 7.21 g L^−1^ d^−1^ in autotrophic and heterotrophic cultivation, respectively^11–13^. Moreover, various carotenoids, such as lutein, β-carotene, violaxanthin, and astaxanthin, accumulate with growth and/or suitable abiotic stress^14^. There are already established species-specific methods for the induction of an axenic culture by serial diluted plating, treatment with antibiotic cocktails, and cryopreservation using a programmed freezing rate and cold shock treatments^15,16^. Moreover, its whole genome sequence has been analysed and annotated accurately^17^. Thus, by establishing a genetic transformation system for *Ettlia* sp. based on the obtained genomic information, engineered strains of *Ettlia* sp. using metabolic engineering could be achieved to elucidate the metabolite biosynthesis mechanisms and to create industrial strains with high potential.

In C*hlamydomonas, Nannochloropsis,* and *Phaeodactylum,* various endogenous promoters have been well established based on published genomic information. A hybrid promoter derived from two constitutively expressed genes, *HSP70A*/*RBCS2* (AR promoter, Heat shock protein 70A-Ribulose bisphosphate carboxylase small chain 2), is one of the most widely used promoters in *Chlamydomonas*. Fucoxanthin-chlorophyll binding protein (FCP), light-inducible protein (LIP), and nitrate reductase (NIT) have been used as inducible promoters for the genetic engineering of *Chlamydomonas* and *Phaeodactylum*^18^. Moreover, the development of a strong novel endogenous promoter based on various omics studies has been carried out for the metabolic engineering of microalgae for stable gene insertion and expression and high-yield production of recombinant proteins. The *Chlamydomonas* RPL23 flanking sequence, which was selected as having uniformly high expression levels based on the analysis of a large set of diurnal transcriptome data, was shown to stably express the *LhcSh* fusion gene (luciferase and zeocin resistance gene) at significantly higher levels than that of AR or the psaD promoter^19^. Promoter HASP1 (highly abundant secreted protein 1) was selected as the most abundant secreted protein based on proteome profiling of the culture medium of *Phaeodactrium tricornutum* using LC–MS/MS analysis, and as a result, its activity on GFP expression was observed during all growth phases^20^. However, because commonly used promoters and transformation conditions have rarely adapted to microalgae, these approaches need to be investigated in interesting strains as species-specific methods. Thus, the development of an endogenous promoter and an optimized expression system is essential for the metabolic engineering of *Ettlia* sp.

To this end, this study used genetic engineering to develop a stable transformation system for the oleaginous microalgae *Ettlia* sp. to improve its industrial applications. The electroporation parameters were determined and a transcriptome analysis was performed to investigate codon usage and highly expressed genes to select strong endogenous promoters. Species-specific expressed vectors were then constructed using two endogenous promoters derived from *CRT* and *psaD* and the terminators, a codon-optimized heterologous protein, and a hygromycin-resistance cassette. Finally, the optimized expression system was verified with transformants using genetic and phenotypic experiments, which showed high gene expression and high repetition.

## Results

### Optimization of electroporation conditions

**Figure 1:**
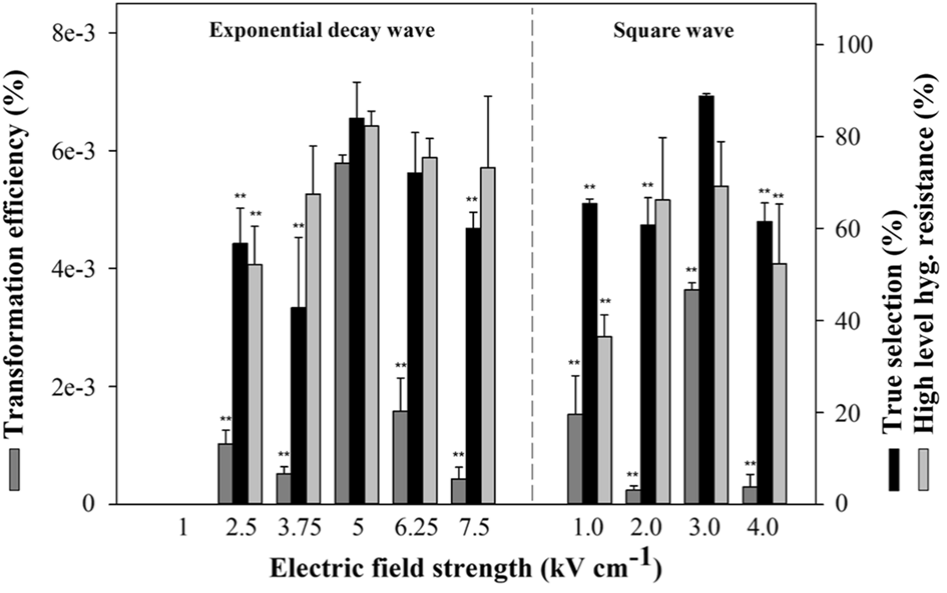
Determination of optimized conditions for electroporation in *Ettlia* sp. by comparison of transformation efficiency, true selection, and high hygromycin resistance with different field strengths and wave types. Asterisks indicate significant differences from the 5 kV cm^−1^ reaction as determined by one-way ANOVA on ranks test (*p* < 0.01). All experiments were performed in triplicate, and the error bar represents the standard deviation (SD) of biological replicates.

The electroporation conditions were optimized using three parameters: waveform, field strength, and number of pulses. As the time constant could not be controlled for an exponential decay wave, it was taken to be approximately 5–15 millisecond (ms), while the square wave was set to 30 ms with two pulses. Positive transformant colonies appeared after 2 weeks on TAP-agar plates containing 100 µg mL^−1^ hygromycin. The transformation efficiency was calculated as the initial selected colonies on the hyg-agar plates per the total number of cells in one reaction. However, since this included background noise, the true selection efficiency (%) was calculated using the cell viability after the 2nd and 3rd transfer, and the result was confirmed by using a microplate reader to detect chlorophyll fluorescence and a colony PCR to amplify the *aphVII* gene (Fig. S1). The ratio of hygromycin-resistant transformants was calculated based on the susceptibility to 50 and 100 µg mL^−1^ hygromycin. When using an exponential decay wave, 5 kV cm^−1^ was the highest transformation efficiency among all the field strengths (Fig. 1), whereas 3 kV cm^−1^ was the highest when using a square wave form with two pulses. No significant relationship was observed between the transformation efficiency and the field strength of either the exponential decay wave or square wave by analysis of Pearson correlation (*p* > 0.05). However, when using an exponential decay wave, a lower field strength did not facilitate pore-mediated penetration of the DNA into the nucleus, showing a low true selection efficiency at 2.5 and 3.75 kV cm^−1^. A similar field strength produced a higher true selection efficiency when using a square wave than when using an exponential decay wave. This stable DNA penetration into the cells may have been induced by the longer duration of the electric shock (30 ms) and the two pulses, even when using a low voltage. The survival of the second selection of transformants was assayed using a high concentration of hygromycin (100 µg mL^−1^) to compare the expression stability of the inserted heterologous gene, *aphVII*. The transformants at 5, 6.25, and 7.5 kV cm^−1^ showed over 80% survival. Even though the wave type affected the transformation efficiency and true selection efficiency, the optimized conditions were finally selected: 5 kV cm^−1^ field strength with an exponential decay wave due to the simplicity of the operation.

### Transcriptome analysis for selecting highly expressed endogenous genes

**Figure 2:**
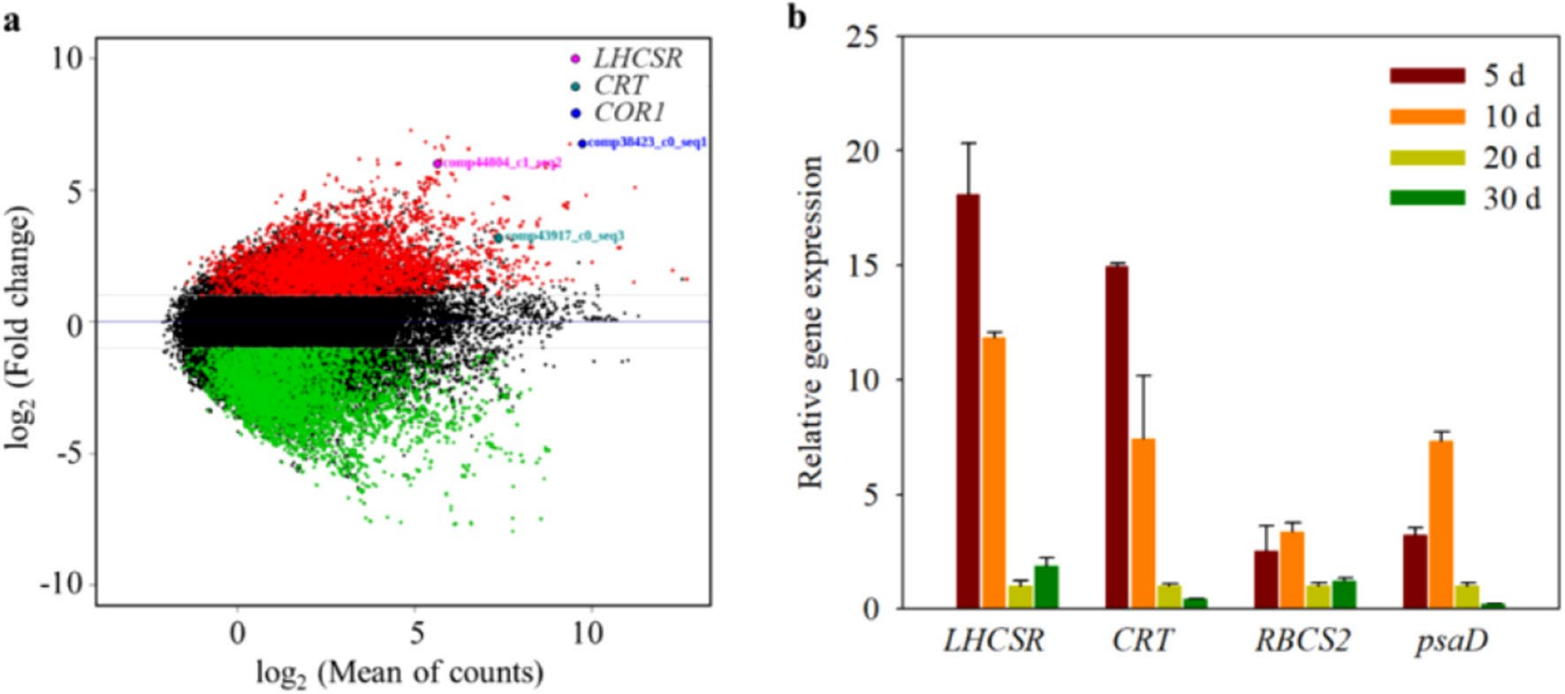
Investigation of three highly expressed genes of *Ettlia* sp. in the late exponential phase versus the late stationary growth phase. These genes were identified by transcriptome data (**a**) and gene expression analysis of four promoter candidate genes by qRT-PCR over time (**b**). Log-scale plot shows mean growth expression level as fragments per kilobase per million reads (FPKM) versus read numbers. Red and blue dots show upregulated and downregulated expression, respectively. qRT-PCR was performed in triplicate.

For stable expression of the inserted gene, regardless of the antibiotic pressure, RNA-sequencing (RNA-seq) was used to identify genes that were highly abundant in the late exponential growth phase in comparison with the late stationary growth phase. The statistics of the transcriptome are summarized in Table 1. A total of 154,808 contigs were assembled using all reads and functionally annotated using three different databases: KEGG for *Chlamydomonas* and NR and UniProt for *Viridaplantae*. Among the annotated contigs (34,898), the number of genes that were differentially expressed by more than twice was estimated at 10,654 (Supplementary Data S1). After a re-evaluation of the read counts, three genes were selected: cold regulation protein 1 (*COR1*, comp38423_c0_seq1), calreticulin (*CRT*, cpom43917_c0_seq3), and light-harvesting complex stress-related (*LHCSR*, comp44804_c1_seq2) (Fig. 2a). Other contigs belonging to these genes also showed a similar expression pattern in the exponential growth phase (Supplementary Data S1). The verification of this expression in the growth stage was analysed by quantitative RT-PCR using mRNA extracted from cultures after 5, 10, 20, and 30 days. Only the *CRT* and *LHCSR* genes matched the RNA-seq results (Fig. 2b). COR1 was excluded, as the BLAST search similarity analysis of the *COR1* gene was too low to annotate the gene, invalidating qRT-PCR. CRT plays a role as a molecular calcium-binding chaperone that promotes folding, oligomeric assembly, and quality control in the endoplasmic reticulum (ER) via the calreticulin/calnexin cycle^21^. This lectin can interact transiently with almost all the monoglucosylated glycoproteins that are synthesized in the ER. Moreover, CRT is also found in the nucleus, suggesting that it may play a role in transcription regulation^22^, resulting in high expression during cell proliferation. The *LHCSR* was also excluded because despite exhibiting high differential gene expression, its expression was lower than that of *CRT*. Moreover, since *LHCSR* is a stress-related protein from the light-harvesting complex (LHC) family that is related to the photoprotective mechanism in microalgae^23^, its use as a promoter is likely limited to autotrophic conditions rather than heterotrophic conditions.

**Table 1:** Transcriptome statistics of strain YC001 in late exponential- and stationary-growth phase.

	Late exponential growth phase	Late stationary growth phase
**Illumina sequencing statistics**		
Number of raw reads	50,658,556	46,807,310
Number of bases (bp)	5,116,514,156	4,727,538,310
Number of reads after trimming	43,726,768	41,404,640
Number of bases after clean up (bp)	3,912,874,708	3,713,880,339
**Assembly**		
Contigs	154,808	
N50	1,901	
Largest contig length (bp)	11,215	
Avg. contig length (bp)	1,060	
Number of bases (bp)	164,216,934	
**Contigs with functional annotation**		
KEGG (*Chlamydomonas reinhardtii*)	10,886	
NR (*Viridiplantae*)	22,111	
Uniprot (*Viridiplantae*)	24,806	
KEGG or NR or GO or uniprot	34,898	
**DEG**		
Number of DEGs (1 < =|log_2_ FC|< = 10)	10,654	
Annotated DEGs (1 < =|log_2_FC|< = 10)	6,406

To compare the efficiency with constitutive promoters, the expression levels of photosystem I protein D (*psaD*) and of ribulose-1,5-bisphosphate carboxylase/oxygenase small subunit II (*RBCS2*), which are used broadly in microalgae, were determined during the growth stage (Fig. 2b). The expression of *psaD* fluctuated more than that of *RBCS2* during the growth stage, although both genes showed high expression throughout. Thus, *CRT* and *psaD* were finally selected to construct the *Ettlia* species-specific expression vector.

### Computational analysis of promoter region within two genes

The gene structure and sequence were confirmed by full-length mRNA sequencing (unpublished data), and the resulting sequences were submitted to NCBI (accession numbers for *CRT* and *psaD* are MN184714, MN184715, and MN184717, respectively). A computational prediction tool was used to define the promoter region as a boundary upstream region of ~ 1.5 kb in each gene and analyse the key promoter elements: cis-acting elements, transcription factor (TF) binding site, and transcription start site. Thus, the promoter region was designed based on the proximal TATA box from the start codon involved with the key elements.

Both promoters exhibited common cis-acting elements, such as CAAT-box, ABRE, E-box, GATA box, SORLIP1AT, and WRKY71, yet not an initiator. ABRE, GATA-box, and G-box elements were found in the binding site of the bZIP TFs, which regulate the stress response. Both promoters included binding sites for the common transcription factors ASF2, MYB, and MYC. While the psaD promoter included a consensus binding site for ASF1 (transcriptional activation by auxin and/or salicylic acid), AP2, and BELL, the CRT promoter interestingly included bZIP and RAV1. The RAV subfamily TFs are more involved in imparting biotic stress tolerance to plants via activation of the *PR* genes. The bZIP family is involved in diverse regulatory functions from carbon metabolism to abiotic and biotic stress tolerance, indicating that the CRT promoter could be constantly activated or inducible against diverse stresses. The psaD promoter included elements related to light responsiveness, such as GT1, GATA-box, I-box, and SORLIP1AT, a component of photosynthesis, indicating that the psaD promoter could be highly expressed during photosynthesis by regulating light (Fig. S2).

The virtual transcription start site of the promoter was determined ~ 400 bp upstream at a start codon based on component analysis of the promoter and full-length mRNA sequencing (Iso-seq). The psaD terminator was also determined ~ 400 bp downstream at a stop codon using Iso-seq and used to construct the vector.

### Codon optimization of cyan fluorescence protein

**Figure 3:**
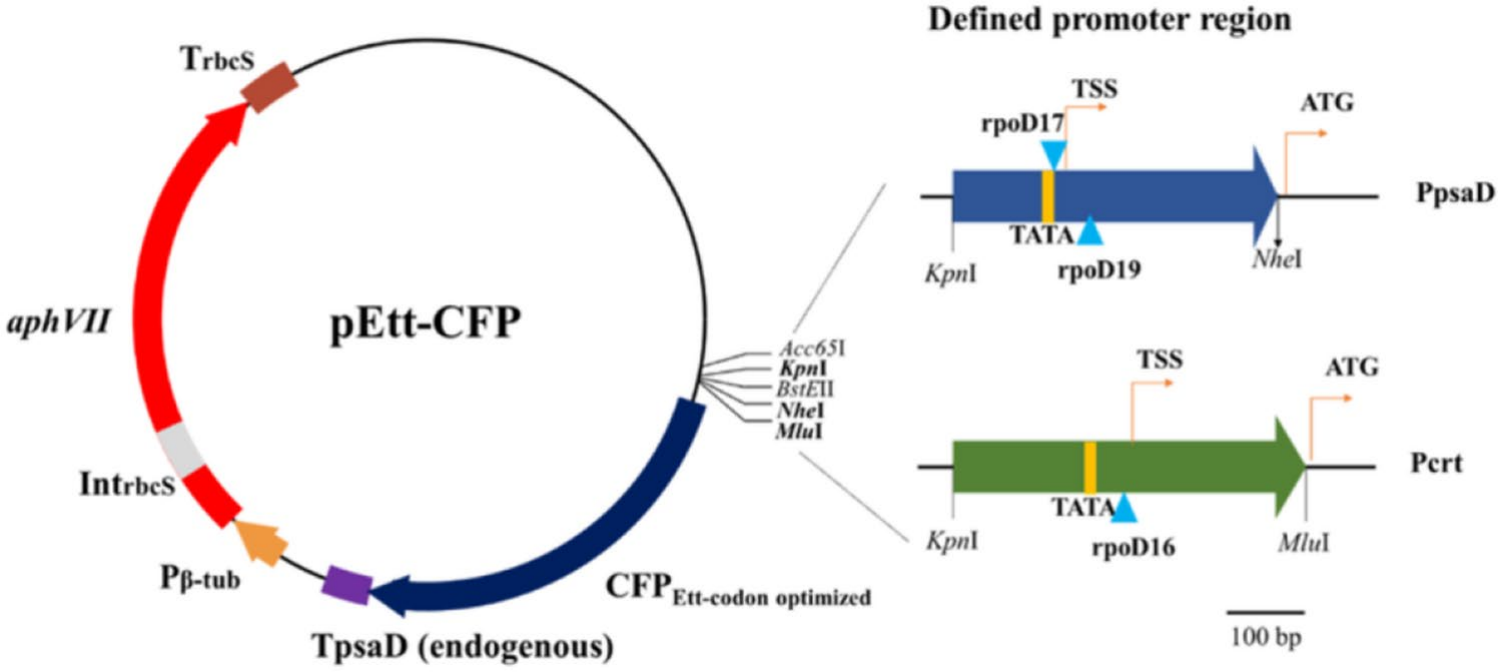
Map of constructed *Ettlia* sp.-specific vectors. pEtt-CFP consisted of two different endogenous promoters originating from the *calreticulin* and *psaD* genes, codon-optimized CFP, an endogenous terminator derived from the *psaD* gene, and a hygromycin-resistant cassette. Transcription factor binding sites were predicted by BPROM (bacterial promoters DB) on the Softberry website and are shown as blue triangles. TSS, transcription start site; P*β*-tub, *β*-tubulin promoter; IntrbcS, first intron in rbcS gene; TrbcS, rbcS terminator.

*Ettlia*-specific vectors were constructed with the following properties: endogenous promoters of CRT and psaD, endogenous terminator of psaD, codon-optimized cyan fluorescence protein (CFP) as a heterologous insertional gene, and a hygromycin-resistant cassette derived from pHyg3 for selection (Fig. 3). To improve protein expression, the codon frequency of *Ettlia* sp. was estimated using numerous transcripts generated by a transcriptome analysis, and the target protein, CFP codon, was optimized according to these results. As the nucleotide preference at the 3rd codon site in *Chlamydomonas* and *Ettlia* sp. was quite different, with the GC contents of 86.2% and 58.4%, respectively, the codon usage bias was clearly distinguishable in each genus (Table S1). While *Chlamydomonas* shows a rather unequal distribution of certain amino acids with a single dominant or minor codon following alanine, glycine, proline, threonine, and valine, the *Ettlia* sp. preference was not concentrated on one codon beyond amino acids and showed a similar pattern to other microalgae: *Chlorella vulgaris, Dunaliella salina, Phaeodactylum triconutum*, and *Volvox carteri* (Supplementary Data S2). While *Chlamydomonas* included 23 rare codons (fraction < 0.1) in 14 amino acids, *Ettlia* sp. included only a few rare codons: leucine (UUA), serine (UCU), and arginine (AGA). Moreover, 4 codons that were rarely used in *Chlamydomonas* were abundantly used in *Ettlia* sp.: threonine (ACA), alanine (GCA), cysteine (UGU), and arginine (CGU). Some of the most dominant codons in *Chlamydomonas* were different in *Ettlia* sp.: glycine (GGC → GGC/GGT), cysteine (TGC → TGT), arginine (CGC → CGT), threonine (ACC → ACC/ACA), and valine (GTG → GTC). According to the properties of codon usage, 70 codons in 7 amino acids among 720 bp of the creCFP gene were altered for optimization in *Ettlia* sp.; 17 transitions (purine–purine) and 53 transversions (purine–pyrimidine) (Fig. S3). The promoters originating from CRT and psaD were combined with the codon-optimized CFP and named pEtt-CRT and pEtt-psaD, respectively.

### Electroporation with constructed vectors

The *Ettlia* sp. electroporation was carried out under optimized conditions. Dozens of transformants were obtained using both pEtt-CRT and pEtt-psaD and were serially transferred onto TAP medium containing 10 µg mL^−1^ hygromycin for genotypic and phenotypic analyses. The initial verification of the insertion vectors was based on colony PCR using an aliquot of the culture, followed by an assay of the susceptibility against various concentrations of hygromycin (10, 20, 50, 100 µg mL^−1^).

Four transformants derived from pEtt-CRT and five derived from pEtt-psaD that showed resistance against 100 µg mL^−1^ of hygromycin were selected, and the insertion into the nucleus was then confirmed with a Southern blot using a CFP probe. Only four transformants were confirmed; these were named crt-17 and crt-18 (generated by pEtt-CRT) and psaD-1 and psaD-5 (generated by pEtt-CRT and pEtt-psaD) (Fig. S4). The unique bands in crt-17 and crt-18 indicated that one copy of the CFP gene was integrated in a different locus, while similar size bands in psaD-1 and psaD-5 and a vague band at the bottom of psaD-5 seemed to show integration of one and/or more than one copy. However, despite resistance to hygromycin in transgenes crt-7, crt-12, psaD-2, psaD-3, and psaD-4, no bands were observed by Southern blot. Transient expression in the transgene can be caused by the position effect, integration of fragmented cassettes, epigenetic-derived transgene silencing, and episomal expression^24–27^. To accurately identify the integration sites of the transgene and validate stable integration, RESDA-PCR was performed, and the obtained bands were cloned to identify each sequence of flanking regions in the integration site. The band pattern of RESDA-PCR was consistent with that of the Southern blot; while a single band was observed in crt-17, crt-18, and psaD-1, two bands appeared in psaD-5. Interestingly, the integration sites of four transformants were indirectly estimated as the 3′-UTR regions of different hypothetical proteins by searching the flanking region sequence in the *Ettlia* genome (Table S2). The 3′-UTR region plays an important role in regulatory processes such as mRNA stability, 3′ end processing and translation. However, according to Shalem et al.^28^, most mutations in the 3′-UTR region are influenced weakly by gene expression except for mutations localized to a single positive TA-rich element. It could be speculated that the localization of integrated heterologous protein in the 3′-UTR region helps to maintain constantly exogenous DNA in the genome without gene silencing because of the decreased effect on the expression of the integrated gene, at least in terms of this *Ettlia* transformation study.

**Figure 4:**
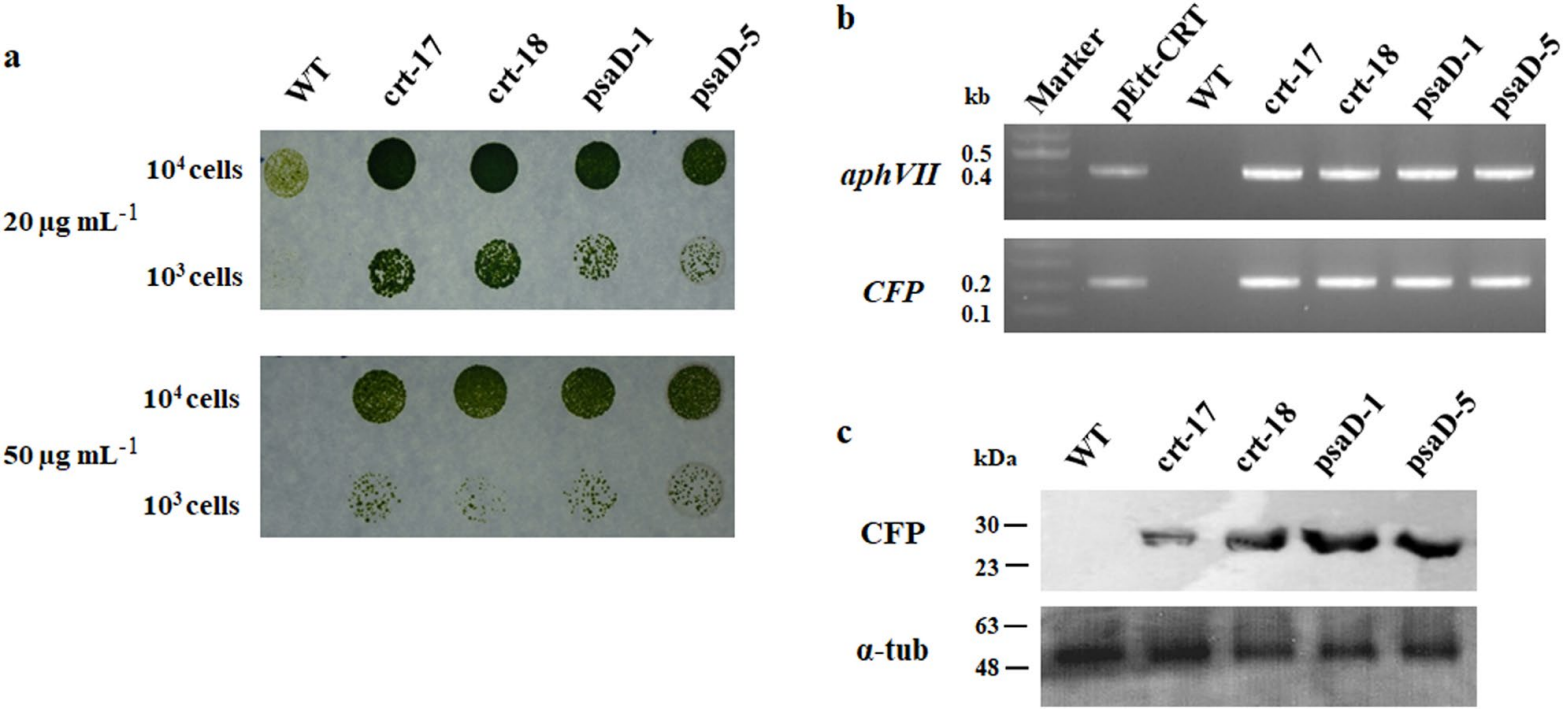
Phenotypic and genotypic verification in transformants generated by pEtt-CRT and pEtt-psaD. Insertion of the vector cassette into the nucleus and stable expression in transformants were confirmed by hygromycin susceptibility (**a**), cDNA amplification (**b**) of the hyg gene, *aphVII* (upper) and *CFP* (bottom), and western blot using CFP probe (**c**). An α-tubulin probe was used as a positive control. WT; wild-type.

**Figure 5:**
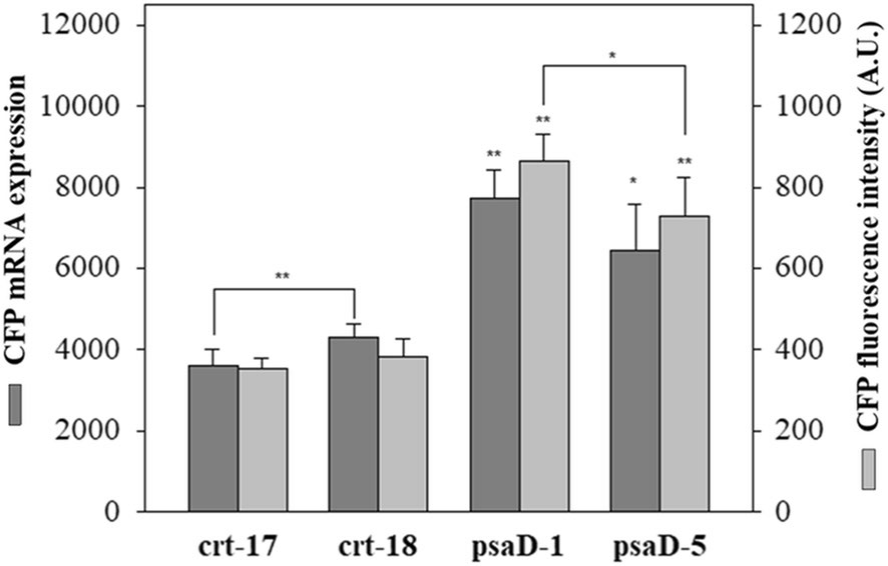
Comparison analysis of two promoter efficiencies in generated transformants by pEtt-CRT and pEtt-psaD. Promoter efficiency was calculated based on CFP fluorescence intensity as determined by qRT-PCR with copy numbers and confocal microscopy with fluorescence intensity values. Asterisks indicate significant differences from crt-17 as determined by Kruskal–Wallis one-way ANOVA on rank test (*p < 0.05, **p < 0.01). Student’s t-test analysis was performed for comparisons with mutant lines (*p < 0.05, **p < 0.01). All experiments were performed in triplicate, and the error bar represents the standard deviation (SD) of biological replicates.

**Figure 6:**
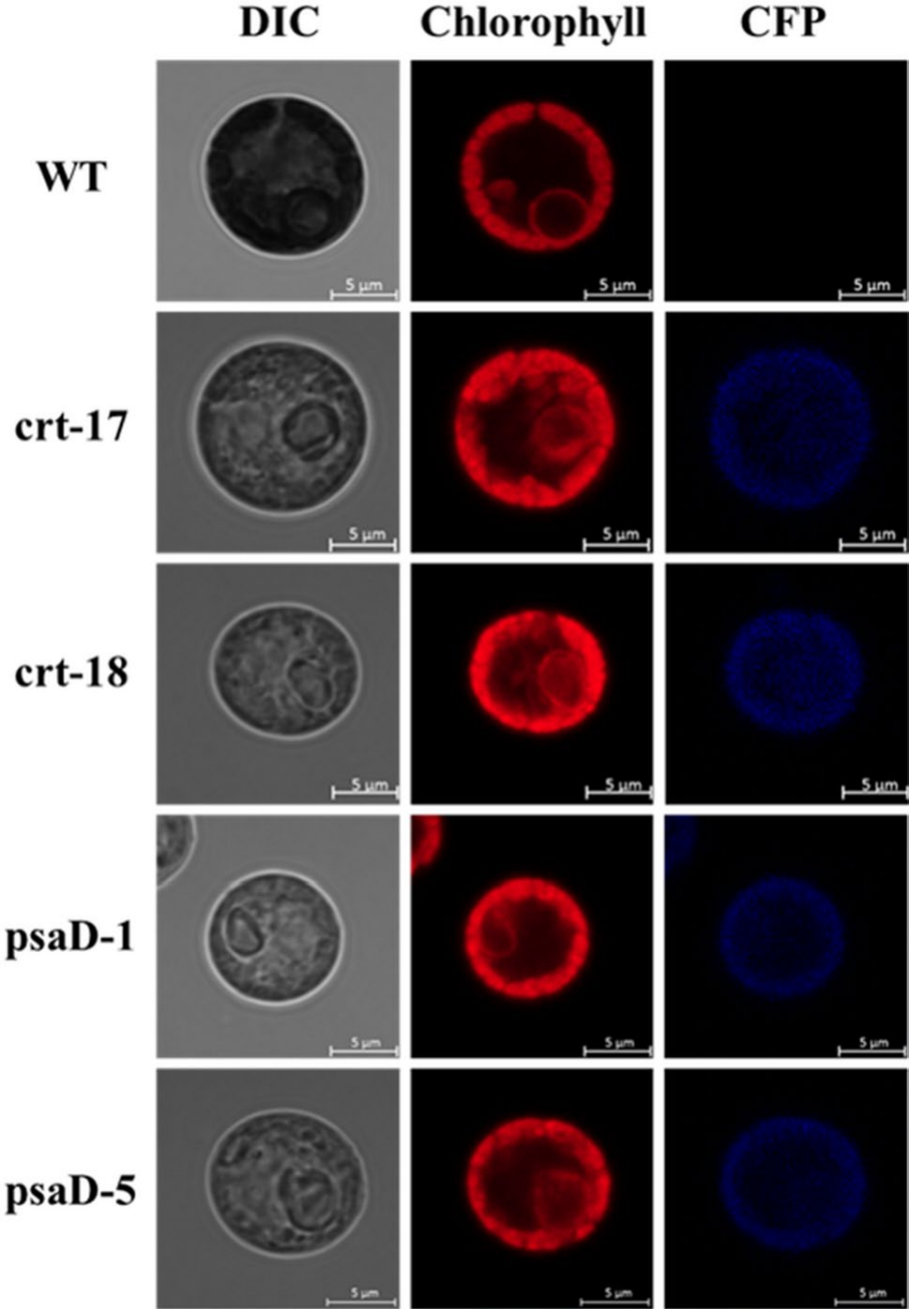
Confocal microscope analysis for measurement of cyan fluorescence in transformants.

PCR using cDNA showed that all 4 transformants clearly amplified both distinct selective markers *aphVII* and *CFP* (Fig. 4b and Fig. S5a) and had a high susceptibility to 50 µg mL^−1^ hygromycin in a liquid culture (Fig. 4a and Fig. S6). The translation of the *CFP* gene in the selected transformants was also accurately identified by the correct size band in a western blot analysis (Fig. 4c and Fig. S5b). The average CFP fluorescence and expression in the transformants were estimated to compare the expression efficiency of the promoters. The expression level of the *CFP* gene in the transformants was found to be consistent with the fluorescence intensity. While the CFP expression level and fluorescence intensity were similar between transformants generated by the same vector, the transformants generated by pEtt-psaD (psaD-1 and psaD-5) showed significantly higher values than the transformants generated by pEtt-CRT (crt-17 and crt-18) (Fig. 5). Although the population shift measured by FACS was more evident in psaD-1 and psaD-5 than in crt-17 and crt-18 (Fig. S7), the cyan fluorescence was similar under a microscope (Fig. 6).

All transformants were transferred to medium with or without hygromycin every 2 months and confirmed by cyan fluorescence and amplification of the *CFP* gene. All were maintained stably for over 20 months.

## Discussion

The progress of transformation using electroporation in *Ettlia* sp. has been optimized based on species-specific characteristics, such as cell wall thickness, cell size, antibiotic resistance, and cultivated tropic preference. Several valid transformation methods have been applied to microalgae, including biolistic particle delivery using glass beads, electroporation, bombardment, and agrobacterium infection. However, such methods cannot be broadly applied to microalgae, except for *Chlamydomonas*. Therefore, this study investigated the use of electroporation to establish the stable transformation of an oleaginous microalga, *Ettlia* sp.

DNA delivery by electroporation is affected by the cell size and rigidity of the cell wall^29^. For example, *Chlamydomonas reinhardtii* and *Monoraphidium neglectum* have a similar cell type to *Ettlia* sp., and their electroporation was previously optimized using a similar voltage to that used in this study^9^. Meanwhile, *Nannochloropsis gaditana* and *Neochloris oleoabundans* have a smaller cell size and require a higher voltage, over 10 kV cm^−1^, or more pulses^8,30^. A higher voltage for an exponential decay wave or a low voltage for a square wave has been shown to be more efficient. For stable nuclear transformation, using the appropriate voltage is critical to reduce the appearance of background noise colonies. Although an in-depth comparison of the *Ettlia* sp. electroporation according to the waveform was not performed in this study, the electroporation efficiency was determined with regard to cell recovery and stable expression in a subculture.

The simple preparation of protoplast and plasmid DNA saves time during the process of electroporation. The protoplast was prepared only for the late exponential growth phase cells as a treatment alternative with a commensal MAX efficiency transformation reagent (Thermo, USA) without autolysin or lysozyme. In *Chlamydomonas*, treatment with autolysin and the use of linear plasmid DNA have been shown to be highly efficient for delivery into the nucleus^31^. However, the preparation of gamete autolysin from *Chlamydomonas* is time-consuming and requires specific expertise. The treatment of restriction enzymes to form linear plasmid DNA also requires optimized treatment and confirmation of the generated form. However, when using the established optimized electroporation conditions for *Ettlia* sp., the delivery efficiency with circular plasmid DNA was similar to that with linear plasmid DNA (data not shown).

According to the antibiotic susceptibility test, *Ettlia* sp. was sensitive to hygromycin, paromomycin, and zeocin concentrations of 100 µg mL^−1^ (Fig. S8). These antibiotics could be replaced with hygromycin in the expression vector as a selection marker to sequentially develop double mutants of *Ettlia* sp. The whole transformation process was carried out using TAP medium to provide mixotrophic conditions. As the growth of *Ettlia* sp. is slightly faster in a heterotrophic and mixotrophic culture than in autotrophic culture^11^, the mixotrophic conditions helped to reduce the preparation time of late exponential cells as protoplast cells and hastened the appearance of resistant colonies on the hygromycin agar plates. However, background noise of under 5% occurred in the transformants and control cells without any plasmid on the hygromycin-agar plates. This occurrence was clearly determined in the subculture to exclude any non-insertional colonies from the nucleus, and the true selection efficiency was recalculated. Some microalgae exist with exogenous DNA in an episomal form in their cells^32^. Thus, for the transformants that lost their hygromycin resistance in the subculture, it was speculated that the insertional plasmid existed transiently as an episome and was easily removed or silenced in the genome during replication. Therefore, these are pointed out as difficult challenges for establishing stable heterologous gene expression in microalgae.

For the stable and strong expression of a heterologous gene in *Ettlia* sp., strong expression promoters need to be determined based on species specificity. In this study, PpsaD and Pcrt were selected as the constitutive promoter and strong endogenous promoter, respectively, according to their expression abundance based on RNA-seq and qRT-PCR, and their activities were compared in terms of the expression efficiency of a foreign gene. While the psaD promoter has already been widely used in microalgae as a constitutive promoter, the development of the CRT promoter for use in microalgae was new. The detection of cyan fluorescence and gene expression in the transformants generated by Pcrt was slightly lower than that in the transformants generated by PpsaD, yet the inserted DNA in both transformants remained stably expressed for over 20 months.

CRT is one of the predominant proteins acting as a molecular calcium-binding chaperone that promotes protein folding by glycoprotein maturation, acts as a buffer, acts as a sensor of intraluminal-free Ca^2+^ levels in the ER, and is related to transcriptional regulation and signal transduction in the nucleus^22^. In *Chlamydomonas*, *CRT* has shown high expression during sexual reproduction in reproductive cells^23^. As CRT plays a role in regulating the stress response, Pcrt may collaborate with abiotic stress to induce more transgene expression, similar to an inducer promoter that is strongly related by lipid metabolism for lipid accumulation. For example, AR promoter, a widely inducible promoter, has been applied to enhance lipid productivity with intermittent heat shock for overexpression via lipid biosynthesis of the lysophosphatidic acyltransferase gene (*LPAAT*) and glycerol-3-phosphate dehydrogenase gene (*GPD1*)^18^. As heat treatment is an abiotic stress that induces changes in lipid composition and lipid contents, it was considered that the transformants may be influenced by combining genetic manipulation and heat shock^33^. To determine the possibility of application as an inducible promoter depending on abiotic stress conditions, the expression level of the CRT gene was investigated under several stress conditions for lipid accumulation: ER stress by DTT treatment (25 µg mL^−1^, 1 h), heat stress (42 ℃, 1 h), and N starvation (24 h). All cells from the wild-type and transgene lines under stressed conditions showed an increase in the CRT gene expression level by three to fivefold compared to the cells in the exponential growth phase (Fig. S9a). These results indicated that Pcrt has potential as an inducible promoter under abiotic stress conditions and can increase lipid productivity in metabolic engineering in combination with stress treatment.

For large-scale protein production in the heterotrophic cultivation of microalgae, the application of an inducible promoter by regulating growth stage has distinct advantages in terms of simplifying the industrial process and enhancing metabolite productivity, especially if a high biomass can be obtained during the early growth stages^34,35^. In the case of *Bacillus subtilis*, Pylb has shown the highest activity for the production of *β*-galactosidase (*bgaB)* during the stationary phase^36^. As Pcrt was found to induce higher expression of the inserted gene during the late exponential and initial stationary growth phases, it could be beneficial to apply mass cultivation to enhance the production of biomass and metabolites simultaneously until the early stationary growth phase. To determine the possibility of application as an inducible promoter by regulation depending on the growth stage in heterotrophic and mixotrophic cultivation, the expression level of the CRT gene was investigated between the exponential growth phase and stationary growth phase. The CRT gene expression patterns in heterotrophic and mixotrophic cultivation were similar, with much higher expression in the exponential growth phase than in the stationary growth phase, and with autotrophic cultivation presenting transcriptome data and qRT-PCR (Fig. S9b). These results implied that Pcrt could play a role as an inducible promoter by regulating the exponential growth phase regardless of nutrient cultivation. Moreover, the optimized conditions for obtaining the maximum biomass productivity in the heterotrophic cultivation of *Ettlia* sp. were recently established using sequential hydrolysis of *Helianthus tuberosus* and algal residue and successfully achieved 43.3 g L^−1^ on day 6 in the early stationary growth phase^11,13^. Thus, Pcrt can be very attractive as an inducible promoter to achieve high production of biomass, lipids, and recombinant proteins within a short period in the case of heterotrophic cultivation.

Regulating the gene expression of *Ettlia* sp. related to lipid and/or starch metabolism, photosynthesis, and transcription factors is essential to improve biomass production and lipid accumulation using the developed vectors. Through the transcriptome analysis in this study, the major metabolizing enzymes of *Ettlia* sp. in glycerolipid biosynthesis, fatty acid biosynthesis, carotenoid biosynthesis, and photosynthesis were determined and summarized with the expression pattern shown in Supplementary Data S3^37^. The identification of the coding domain sequence of key enzymes in metabolic pathways is an essential prerequisite to regulate the gene expression level in metabolic engineering approaches. By modifying these genes, commercial strains that are suitable for application in several industries could be developed soon.

In further research, the optimized electroporation conditions and developed vectors could also be used to deliver genome editing components, such as ZFNs, TALEN, and CRISPR/Cas9. In certain microalgae, the stable application of CRISPR/Cas9 has been reported to regulate the metabolic pathway^5^. However, a remaining challenge is the delivery and stable expression of gRNA and Cas9 in cells. The construction of a plasmid for the stable expression of gRNA and Cas9 requires an endogenous promoter and codon optimization of the Cas9 protein. In this case, pEtt-CRT and pEtt-psaD could easily be applied to construct plasmids^38–40^. A ribonucleoprotein (RNP) is currently preferred over a plasmid for genome editing due to its low toxicity, low off-target effects, and easy preparation without plasmid construction. Using optimized electroporation conditions, RNP could be used for delivery into *Ettlia* sp. cells. Although the delivery efficiency differs depending on the materials, such as DNA and RNP^39^, the success of generating a knock-out mutant using electroporation with RNP in *Chlamydomonas* has already been reported, and the concentration and molar ratio of the mixture of gRNA and Cas9 have been considered. Thus, the application of rapid advances in genome editing technology to *Ettlia* sp. cells using the expression system developed in this study can contribute to microalgae industrial production with high value compounds.

## Methods

### Strain and culture conditions

*Ettlia* sp. was obtained from the Korea Collection for Type Cultures (KCTC 12109), cultivated on a tris–acetate-phosphate (TAP) agar plate for 12 days, and transferred to TAP liquid medium for the mid-log phase at 25 ± 1 °C, 120 µmol m^−2^ s^−1^. To select the transformants, the cells were cultivated in liquid and/or agar TAP medium containing hygromycin 5, 10, 20, 50, and 100 µg mL^−1^. For heterotrophic culture, *Ettlia* sp. was cultivated on BG-11 medium containing a carbohydrate source (30 g L^−1^ fructose and 1.5 g L^−1^ yeast extract) at 35 °C until the stationary phase without light. For the induction of ER stress or heat stress, exponential phase cells were treated with dithiothreitol (final conc. 25 µg mL^−1^) for 1 h or incubated at 42 ℃ for 1 h. For nitrogen starvation, exponential phase cells were harvested and washed three times with BG-11 medium without nitrogen (BG11-N) before incubation in BG11-N. The cells that were transferred to BG11-N medium were incubated for 1 day. The cells were simultaneously cultivated in sets of three flasks for biological replicates, and each flask was harvested for analysis. All experiments were performed in triplicate.

### Antibiotic susceptibility

The susceptibility of *Ettlia* sp. to antibiotics was monitored on BG-11 agar plates. A total of 10^4^, 10^5^, 10^6^, and 10^7^ cells were dropped on BG-11 agar medium with various concentrations of hygromycin, paromomycin, and zeocin (final conc. 5, 10, 20, 50, 100 µg mL^−1^). The plates were incubated at 25 ℃ with 120 µmol m^−2^ s^−1^ for 2 weeks.

### Optimization of electroporation conditions

Protoplasts were prepared from cells in the late exponential growth phase (1 × 10^7^ cells mL^−1^) that had been cultured in TAP medium for 5 days. The harvested cells were washed with distilled water to remove the medium and then washed twice with 10 mL of a MAX efficiency transformation reagent (Invitrogen, USA). The cells were resuspended in the transformation reagent at a concentration of 1 × 10^8^ cells mL^−1^. To 250 µL of the cell suspension, 2 µg of linear formed-vectors pCr102^41^ restricted by ScaI (Enzynomics, Korea) was added, and the mixture was incubated at 4 °C for 10 min.

Electroporation was performed using a Gene Pulser Xcell (Bio-Rad, USA) to investigate three parameters: waveform, field strength, and number of pulses. The other parameters were fixed as follows: capacitance 50 µF, resistance 800 Ω, preincubation of mixture at 4 °C, 2 µg of linear plasmid DNA, and a 2 mm gap size in the cuvette length (Table 2). All experiments were performed in triplicate. Two types of waveforms were used: exponential decay waves and square waves. The field strength (kV cm^−1^) was varied from 100 V to 1,250 V. For the number of pulses, two pulses occurred at 5 s intervals when using a square wave.

**Table 2:** Electroporation conditions with various field strength and wave forms used in this study.

Wave form	Field strength (kV cm^−1^)	Direct voltage (V)	Capacitance (µF)	Resistance (Ω)
Exponential decay wave	1	200	50	800
	2.5	500		
	3.75	750		
	5	1,000		
	6.25	1,250		
	7.5	1,500		
Square wave	1	200	–^a^	–^b^
	2	400		
	3	600		
	4	800		

^a^Number of pulses: 2, Pulse interval: 5 s.

^b^Not controlled.

The prechilled mixture was transferred to an ice-cold cuvette immediately before electroporation. After electroporation, the cells were transferred into 20 mL of TAP liquid medium containing 60 mM sucrose for 16 h and spread on a TAP agar plate containing 100 µg mL^−1^ hygromycin. After 2 weeks, the hygromycin-resistant cells were transferred to liquid TAP medium containing 20 μg mL^−1^ hygromycin. For the selected mutants, the insertion of *aphVII* and *CFP* was confirmed using colony PCR with the appropriate primers and by measuring the chlorophyll intensity using a microplate reader (BioTek, USA) with excitation at 652 nm and emission at 668 nm. All the primer sequences used in this study are listed in Table S3.

Statistical analysis was carried out in R version 4.0.0. Correlation analysis between true selection, hygromycin resistance, and electric field strength was performed using Pearson correlation analysis, and transformation efficiency was estimated using a one-way ANOVA on rank test.

### Transcriptome and iso-seq analysis

Total RNA was isolated from *Ettlia* sp. cells in the late exponential growth phase and late stationary growth phase that had been cultured in BG-11 medium under 120 µmol photons m^−2^ s^−1^ at 25 °C for 14 days and 30 days, respectively. The harvested cells were rapidly frozen using liquid nitrogen, and the cell walls were disrupted using a mortar and pestle. After the grinding step, the RNA was extracted using TRIzol (Invitrogen, USA) and an RNeasy Mini Kit (Qiagen, USA). The integrity of the extracted RNAs was checked using an Agilent 2100 Bioanalyzer (Agilent Technologies, USA) based on the following criteria: RNA 28S/18S ratios higher than 1.0, RNA integrity numbers (RIN) ≥ 8.5, and at least 3 µg of total RNA (≥ 300 ng µL^−1^).

The library construction and sequencing were performed using the Illumina HiSeq 2500 platform (2 × 101 cycles) at KRIBB. Briefly, the cDNA library was constructed using a TruSeq RNA Sample Prep Kit v2 with ribosomal RNA removed from the samples (without biological replicates). Using the CLC Genomics Workbench, paired reads were imported and first quality-trimmed with the below quality values: low quality limit 0.01, max 1 ambiguous nucleotide allowed per read, min length 50 nt^42^. The trimming of paired data was subjected to RNA-seq analysis v2.1 using PGAP annotation. Quantile normalization was selected for genes with 1 or more fragments per kilobase million (FPKM) in both samples, and the analysis of differentially expressed genes (DEGs) was carried out by a proportion-based test.

### Promoter prediction and vector construction

A 1.5 kb region upstream of the CDS was targeted for predicting the promoter site and computationally predicted using FGENESH (Softberry, https://linux1.softberry.com/berry.phtml). The location and distribution of putative cis-acting elements in promoters were analysed using PLACE (https://sogo.dna.affrc.go.jp), PlantCARE (https://bioinformatics.psb.ugent.be), and FGENESH (https://linux1.softberry.com/berry.phtml).

The endogenous promoter::codon-optimized CFP::TpsaD cassette was created following a traditional cloning protocol. The codon-optimized CFP was synthesized at Bioneer (Daejeon, Korea) and replaced with creCFP in pCr102. The codon usage was generated from the 156,858 contigs from RNA-seq and adjusted for codon optimization of the creCFP derived from pCrCFP based on one amino acid with one codon using an optimizer tool (https://genomes.urv.es/optimizer). Other microalgal codon usage was obtained from the HIVE database (https://hive.biochemistry.gwu.edu/review/codon). The endogenous terminator of psaD in *Ettlia* sp. was defined as an ~ 420 bp region behind the CDS and was ligated with the end of the CFP region at the *Hind*III/*Nde*I sites. The sequences of two endogenous promoters, Pcrt and PpsaD, were amplified with gDNA and subcloned upstream of CFP::TpsaD at the *Kpn*I/*Mlu*I or *Nhe*I sites, respectively. All primer sequences are listed in Table S3.

### Southern blot assay

The DNA of the transformants was extracted using a modified phenol–chloroform method and digested with BamHI (Enzynomics, Korea) for 16 h. The fragmented DNA was then separated on 0.8% agarose gels at 35 V for 16 h. After separation, the gel was washed with denaturation buffer (3 M NaCl, 0.4 M NaOH) and transfer buffer (3 M NaCl, 8 mM NaOH)^6^. The DNA fragments were transferred onto Hybond N + membranes (Amersham Biosciences, Sweden) by capillary transfer. UV cross-linking was then performed. A 220 bp probe was amplified with F6 and R226-CFP primers and labelled with ^32^P. After performing the hybridization at 65 °C for 16 h, the membrane was washed with SSC buffer and exposed to X-ray film for 3 days.

### Quantitative real-time PCR analysis

RNA was extracted using an RNeasy Mini Kit (Qiagen, USA) after culturing in BG11 medium under 120 µmol photons m^−2^ s^−1^ at 25 °C for 5, 10, 20, and 30 days. Complementary DNA (cDNA) was synthesized from the total RNA by reverse transcriptase (Promega, USA). qPCR was performed with SYBR green (Bio-Rad, USA) using a CFX Connect Real-Time System (Bio-Rad, USA). The level of CFP expression was calculated using absolute and relative quantification methods to generate a standard curve with pEtt-CRT and 2^−ΔΔCT^ methods using a housekeeping gene (GAPDH), respectively. The plasmid DNA was serially diluted from 30 copies (1.25 fmol) to 300,000 copies (1.25 nmol) and then amplified with F6 and R226-CFP primers to establish a standard curve. The slope score was − 3.35. The expression level of the *CFP* gene was calculated according to the cDNA concentration using the Ct value. Three biological replicates were analysed, and three technical repeats were performed per sample.

### Restriction enzyme site-directed amplification PCR (REDA-PCR)

The site on the plasmid with the random insertion was confirmed by RESDA-PCR^43^. The PCR conditions were as described in the Kong and Li-Beisson^44^. The 1st PCR was amplified in a 20 µL volume using Taq DNA polymerase (Biofact, Korea) with degenerate primers and pEtt-F1 primers. The 2nd PCR was carried out in a 20 µL volume using Platinum II Taq Hot-Start DNA polymerase (Invitrogen, USA). The Q0 and pEtt-F2 primers were used for amplification in the 2nd PCR. The final PCR product was separated on a 1% agarose gel at 100 V for 30 min using electrophoresis. The amplified band was extracted by Wizard SV gel and a PCR clean-up system (Promega, USA). PCR fragments were cloned using the All in One PCR Cloning Kit (Biofact, Korea), cultured in LB medium with kanamycin and ampicillin (final conc. 50 µg mL^−1^), and sequenced with M13 universal primers.

### Western blot assay

To confirm the expression of the *CFP* gene at the protein level, western blotting was performed. The cells were broken by sonication based on 3 cycles of on/off (1 s/3 s). After sonication, the disrupted cells were boiled with 4× sample buffer (Invitrogen, USA) for 10 min and loaded on an SDS-PAGE gel. After separation, the proteins were transferred to polyvinylidene fluoride (PVDF; Bio-Rad, USA) membranes by a Mini Trans-Blot system (Bio-Rad, USA) and incubated in a 5% (w/v) skim milk solution in Tris-buffered saline containing Tween-20 (TBST) for blocking. The primary and secondary antibodies were GFP rabbit polyclonal IgG (Thermo Fisher Scientific, USA) and goat anti-rabbit IgG HRP (Abcam, UK), respectively. The fluorescence signals of the CFP protein-antibody complex in the PVDF membrane were reacted with a western ECL peroxide solution and Luminol/Enhancer solution (Bio-Rad, USA). The membrane was exposed in a Vivid X-ray developer and rapid fixer (Dong-jin Corperation, South Korea), and the complex was then detected using X-ray film (AGFA, Belgium).

### Confocal and fluorescence-activated cell sorting (FACS) analysis

To visualize CFP expression in the cells, the transformants were observed under a confocal laser scanning microscope (Carl Zeiss AG, Germany). The excitation and emission wavelengths of the CFP were 458 nm and 480 nm, respectively. The excitation wavelength of chlorophyll autofluorescence is 655 nm; the emission wavelength was detected between 650 and 700 nm. Fluorescence-activated cell sorting (FACS, BD Bioscience, USA) was used to investigate the distribution of the transformants according to their cyan-fluorescence. Twenty thousand cells of each transformant were screened using violet-A (ex. 407 nm, em. 470 nm) and preCP-cy5-5-A (ex. 630 nm, em. 680 nm) and compared with live CFP-expressing cells. All analyses were performed in three biological replicates.

## Supplementary information

Supplementary Legends.

Supplementary Data S1.

Supplementary Data S2.

Supplementary Data S3.

Supplementary Information.

## Data Availability

The BioProject accession number for the transcriptome sequencing is PRJNA554532. All the sequencing raw data files are available from the Sequence Read Archive under accession number SRX6448205 and SRX6448206.
