# Supplementary material for: Development of a species-specific transformation system using the novel endogenous promoter calreticulin from oleaginous microalgae Ettlia sp
Source: Sci Rep. 2020 Aug 18;10:13947. doi: 10.1038/s41598-020-70503-2 (PMC7434781; doi:10.1038/s41598-020-70503-2)
Supplement: Supplementary file 1 — Supplementary Legends. [file 41598_2020_70503_MOESM1_ESM.docx]

**Supplementary Information**

**Figure S1** Screening of high-potential transformants showing high resistance against hygromycin using a microplate reader with an intensity of chlorophyll fluorescence among hundreds of transformants generated by 3 kV cm^-1^ ((a) and (c)) and 5 kV cm^-1^ ((b) and (d)).

**Figure S2** Location of putative cis-acting elements in two endogenous promoters. The start codon (ATG) is marked in bold red. rhoD; transcription factor binding sites.

**Figure S3** Codon optimization of cyan fluorescence protein.

**Figure S4** Southern blot analysis for detecting inserted vectors with a CFP-probe in the digested transformant genome generated by pEtt-Pcrt (a) and pEtt-PpsaD (b).

**Figure S5** The full-length images for Phenotypic and genotypic verification in transformants by using cDNA amplification (a) and western blot (b).

**Figure S6** Hygromycin susceptibility of two types of transformants generated by pEtt-Pcrt (a) and pEtt-PpsaD (b).

**Figure S7** FACS analysis for observing the shift in cyan fluorescence in transformants.

**Figure S8** Antibiotic susceptibility of *Ettlia* sp. to paromomycin (a) and zeocin (b).

**Figure S9** Quantitative analysis of *CRT* gene expression under abiotic stress (a) and dependent on nutrient cultivation (b).

**Table S1** Summarized information on GC contents of the genome and codons of *Ettlia* sp. and chlorophyte strains.

**Table S2** Identification of the integration site in four transformants of *Ettlia* sp.

**Table S3** Sequence information on primers used in this study.

**Data S1** List of DEGs between the late exponential and stationary growth phases.

**Data S2** Codon usage frequency with *Ettlia* sp. and chlorophyte strains.

**Data S3** List of the major metabolic genes and their expression levels in the late exponential and stationary growth phases in glycerolipid biosynthesis, fatty acid biosynthesis, carotenoid biosynthesis, and photosynthesis.
