# Supplementary material for: Development of a species-specific transformation system using the novel endogenous promoter calreticulin from oleaginous microalgae Ettlia sp
Source: Sci Rep. 2020 Aug 18;10:13947. doi: 10.1038/s41598-020-70503-2 (PMC7434781; doi:10.1038/s41598-020-70503-2)
Supplement: Supplementary file 5 — Supplementary Information. [file 41598_2020_70503_MOESM5_ESM.pdf]

Supplementary Data

Development of a species-specific transformation system using the novel endogenous promoter calreticulin from oleaginous microalgae *Ettlia* sp.

Jun-Woo Lee[1,2], Min-Woo Lee[1,3], Ji-San Ha[4], Dae-Soo Kim[5], EonSeon Jin[2], Hyung-Gwan Lee[1,3*], and Hee-Mock Oh[1,3*]

[1] Cell Factory Research Center, Korea Research Institute of Bioscience and Biotechnology (KRIBB), Daejeon, Republic of Korea

[2] Department of Life Science, Hanyang University, Seoul, Republic of Korea

[3] Department of Environmental Biotechnology, University of Science and Technology, Daejeon, Republic of Korea

[4] Department of Biological Sciences, Sungkyunkwan University, Suwon, Republic of Korea

[5] Rare Disease Research Center, Korea Research Institute of Bioscience and Biotechnology (KRIBB), Daejeon, Republic of Korea

[*]Correspondences:
Dr. Hyung-Gwan Lee
trustin@kribb.re.kr

Dr. Hee-Mock Oh
heemock@kribb.re.kr

Table S1. Summarized information on GC contents of the genome and codons of *Ettlia* sp. and chlorophyte strains.

Table S2. Identification of the integration site in four transformants of *Ettlia* sp.

Table S3. Sequence information on primers used in this study.

Table S1. Summarized information on GC contents of genome and codons with *Ettlia* sp. and chlorophyte strains

Genus/Species	Number of CDSs (codons)	GC (%) in codon			
		Total	1st	2nd	3rd
Botryococcus	63 (28,063)	55.16	57.37	42.27	65.87
Chlamydomonas reinhardtii	14,410 (655,6219)	68	68.49	52.34	83.16
Chlorella	9,780 (4,456,470)	68.86	70.59	51.33	84.67
Dunaliella	374 (164,426)	57.68	58.77	43.06	71.26
Ettlia sp. YC001	22038 (unpredicted)	55.34	61.43	46.15	58.44
Haematococcus lacustris	98 (40,753)	60.09	61.78	44.36	74.11
Nannochloropsis	3554 (1,057,813)	58.27	61.18	46.59	67.04
Phaeodactylum tricornutum	10,408 (4,843,550)	51.06	56.48	43.67	53.04
Scenedesmus	23 (5,278)	58.99	62.47	43.2	71.31
Volvox carteri	14,436 (8,215,986)	62.98	66.11	52.34	70.51

Table S2. The information of the integration site in four transformants of *Ettlia* sp.

Mutant	Location	Inserted position	Transcript name	Annotation
crt17	scaffold153-3	3UTR	comp46443_c0_seq2	hypothetical protein
crt18	scaffold2505-3	3UTR	comp47073_c0_seq1	phototropin
psaD1	scaffold771-10	3UTR	c15672_f1p6_2824	hypothetical protein
psaD5	scaffold210_5	3UTR	-	hypothetical protein
	scaffold433-12.5	3UTR	comp46587_c1_seq7	Predicted protein

Table S3. Sequence information on primers used in this study

Target	Primer name	Sequence	Usage
aphVII	F1204-aphVII	CCATTCCGAGGTCTTCCCGGAACTGCT	Confirmation of transformants
	R1599-aphVII	GGTCTCCTCGAACACCTCGAAGT	
CFP	F6-cfp	TCAACGGCCACAAGTTCTCGGTCT	Confirmation of transformants<br>Southern blot probe
	R226-cfp	CGTCCAGTGTTTCGCACGTTACC	
psaD	F3-psaD	CGCTTGTGCTGCTAACCGCTA	Vector construction
	R3-psaD	CGACAAACCGGAAGCCGATCA	
	F319-psaD-RT	cgCAGGTGGAGGAGTTCTATGT	qRT-PCR
	R511-psaD-RT	GGTACACCCTGTAGAAGCATGGctg	
CRT	F-ett-CRT-pm	CAATAGCGCTCAGTGGTTGA	Vector construction
	R-ett-CRT-pm	CAACAACCCACTGATCGCAGT	
	F388-CRT-RT	gcGAGACCCCATACTCCATCATGT	qRT-PCR
	R503-CRT-RT	cgCACTTGATGTCCTTTTTGACCA	
LHCSR	F236-Lhcsr	cgCTGGAGGGAGTCTGAGATTGTT	qRT-PCR
	R435-Lhcsr	cagAGACCAATAGCCAGCAAGAGAG	
RBCS2	F159-rbcs2	cgCCAGGTCGACTACATTGTGAACa	qRT-PCR
	R297-rbcs2	catTGTCCAGTACCTGTTGTCGAAGt	
GAPDH	F369-GAPDH	AGAGATGGAGATTGACCTCG	qRT-PCR
	R549-GAPDH	GATCTTGTCGCTGTGCTTGT	

Degeneration of integration site in transgene	DegBamHI	CCAGTGAGCAGAGTGACGIIIIINSGGATCCW	RESDA-PCR
	DegPstI	CCAGTGAGCAGAGTGACGIIIIINSCTGCAGW	
	DegKpnI	CCAGTGAGCAGAGTGACGIIIIINSGGTAACW	
	Q0	CCAGTGAGCAGAGTGACG	
aphVII	pEtt-F1	CGCTCGTTGATCTGAGCCTTGCC	
	pEtt-F2	GATACTGCTCTCAAGTGCTGA	

Figure S1. Screening of transformants using microplate-reader with intensity of chlorophyll fluorescence under various hygromycin concentrations. Detection used excitation at 652 nm and emission at 668 nm, and was normalized by OD_{680}. (a) and (b) show the results for 20 µg mL^{-1} of hygromycin, and (c) and (d) are for 50 µg mL^{-1}. (a) and (c) are the results for 3 kV cm^{-1}, and (b) and (d) are for 5 kV cm^{-1}. The fluorescence intensity was about 1.5 times higher with 5 kV cm^{-1}. The transformants that showed high growth in 50 µg mL^{-1} were selected for genomic and phenomic confirmation.

Figure S2. Location of putative cis-acting elements in psaD promoter (a) and CRT promoter (b). The start codon (ATG) is marked in bold red. rhoD; transcription factor binding sites

Figure S3. Codon optimization of cyan fluorescence protein based on one amino acid taking one codon. The sequence length was 720 nucleotides, which showed 650 matches, 17 transition changes (Purine ↔ Purine / Pyrimidine ↔ Pyrimidine), and 53 transversion changes (Purines ↔ Pyrimidines) between the query and optimized sequence. The alignment analysis was carried out by using BioEdit Ver. 6.0.[1]

Figure S4. Southern blot analysis for detecting inserted vectors with a CFP-probe in the digested transformants genome generated by pEtt-Pcrt (a) and pEtt-PpsaD (b). WT; wild-type

Figure S5 The full-length images for Phenotypic and genotypic verification in transformants by using cDNA amplification (a) and western blot (b).

Figure S6. Hygromycin susceptibility of two types of transformant generated by pEtt-Pcrt (a) and pEtt-PpsaD (b). WT; wild-type

Figure S7. FACS analysis for observing the shift in cyan fluorescence in transformants: comparison of same gate designed by forward scatter (FSC) and side scatter (SSC). WT; wild-type

Figure S8. Antibiotic susceptibility of *Ettlia* sp. to paromomycin (a) and zeocin (b)

Figure S9. Quantitative analysis of *CRT* gene expression under abiotic stress (a) and dependent on nutrient cultivation (b). WT, wild-type; M.E., mixotroph exponential phase; M.S., mixotroph stationary phase; H.E., heterotroph exponential phase; H.S., heterotroph stationary phase.

Figure S1.

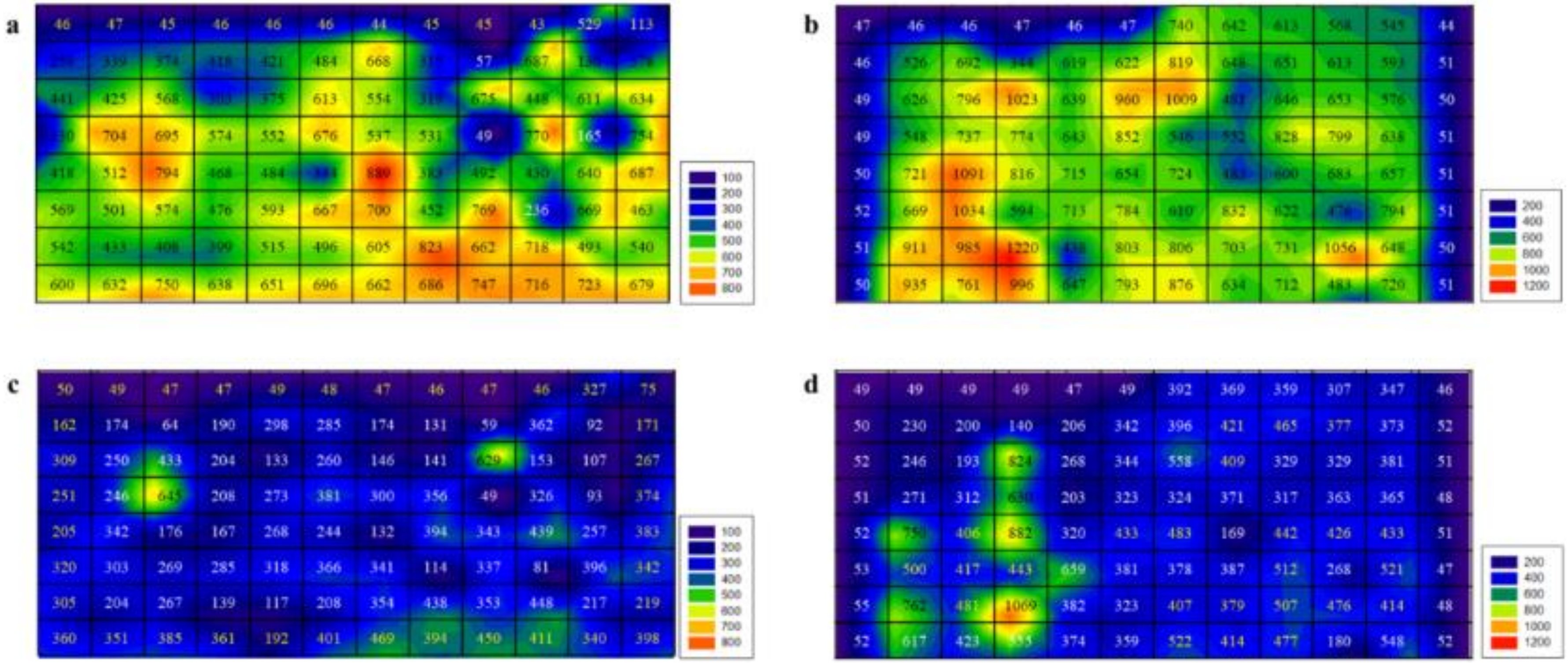
a
100
200
300
400
500
600
700
800
b
200
400
600
800
1000
1200
c
100
200
300
400
500
600
700
800
d
200
400
600
800
1000
1200

Figure S2.

(a) psaD promoter

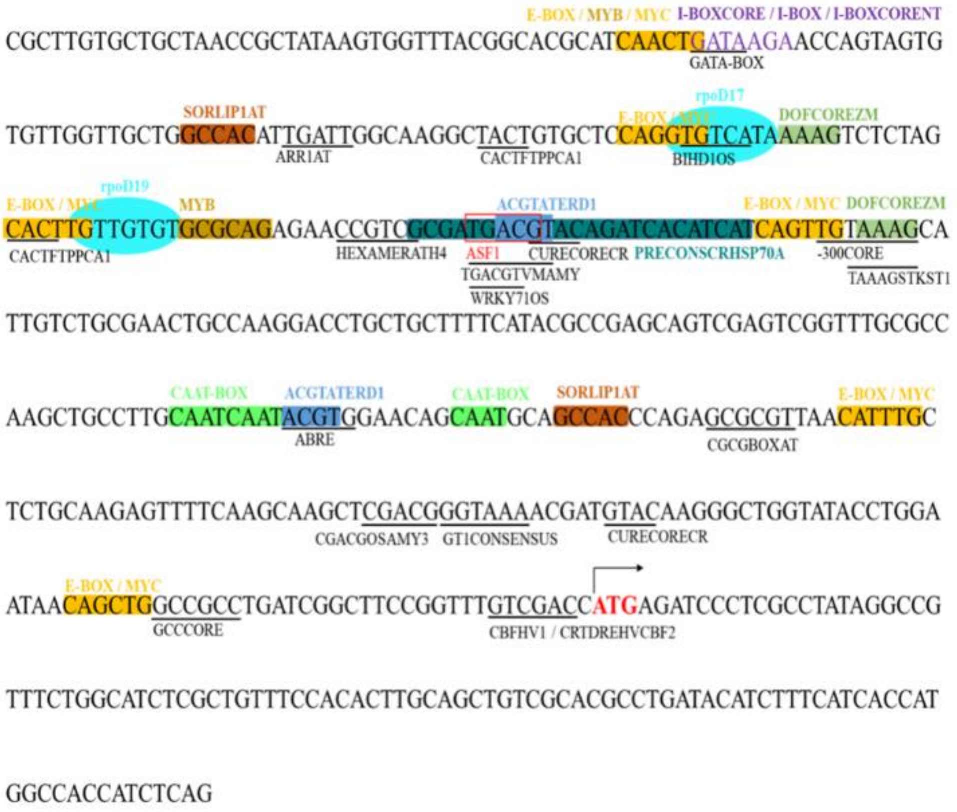

(b) CRT promoter

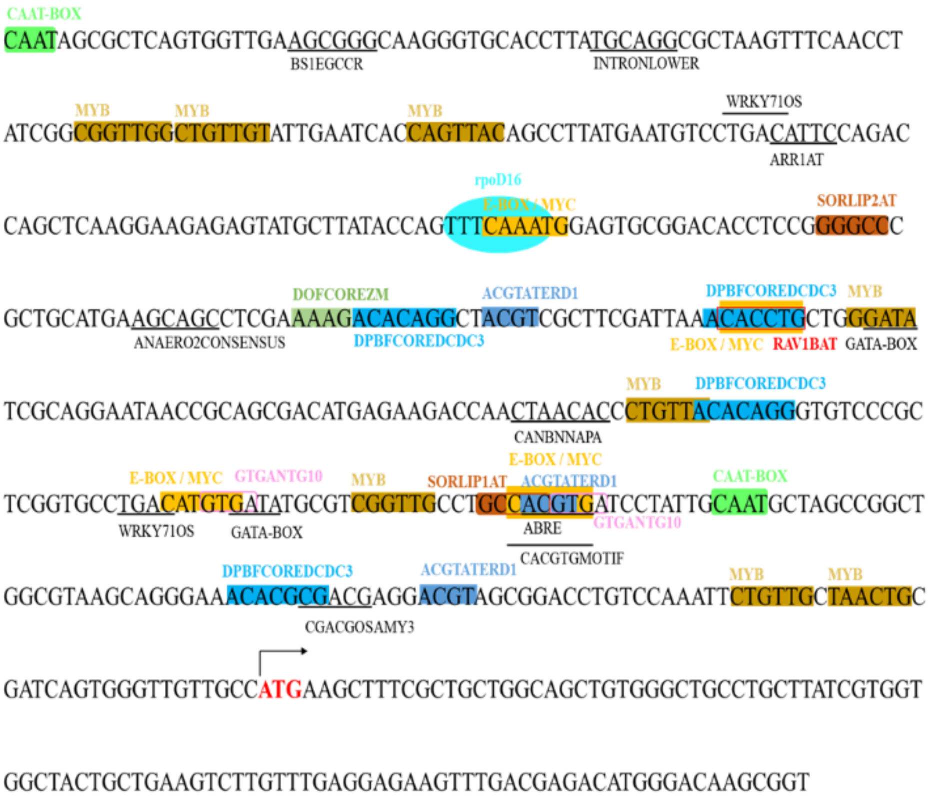

Figure S3.

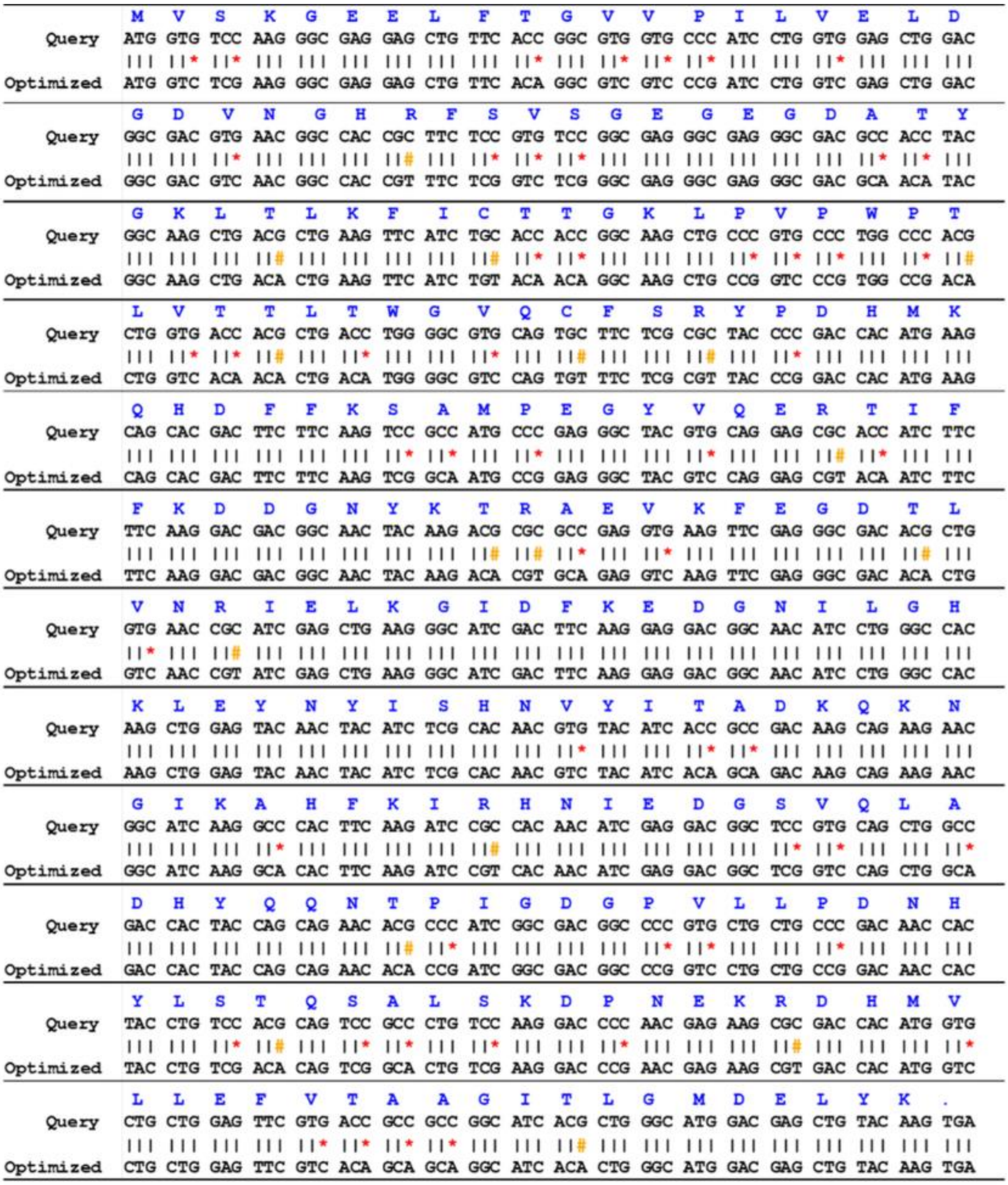

Figure S4.

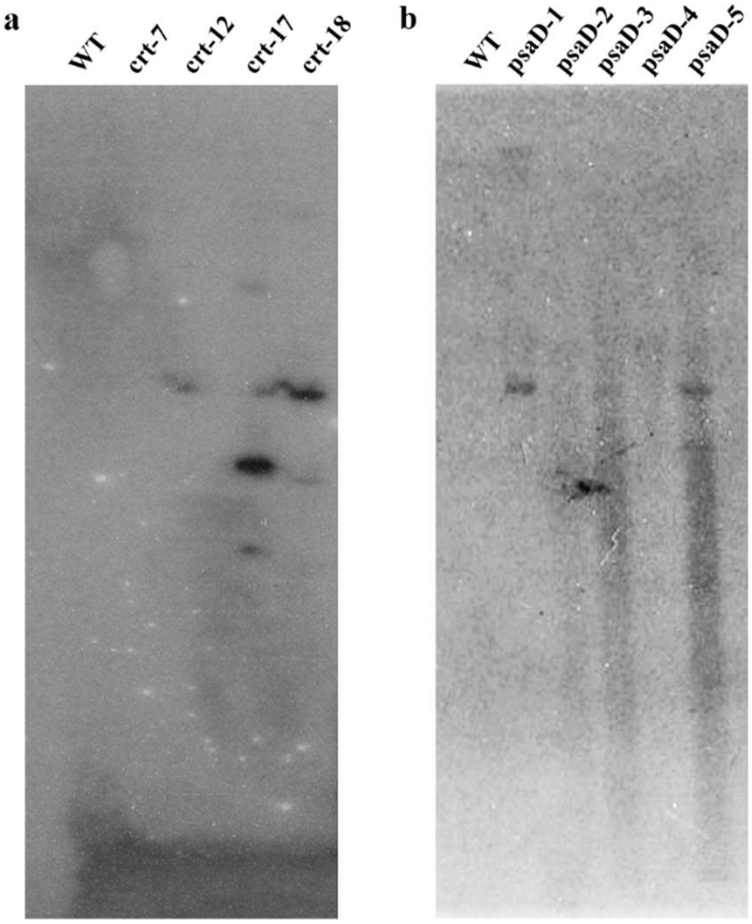
a
WT
crt-7
crt-12
crt-17
crt-18
b
WT
psaD-1
psaD-2
psaD-3
psaD-4
psaD-5

Figure S5.

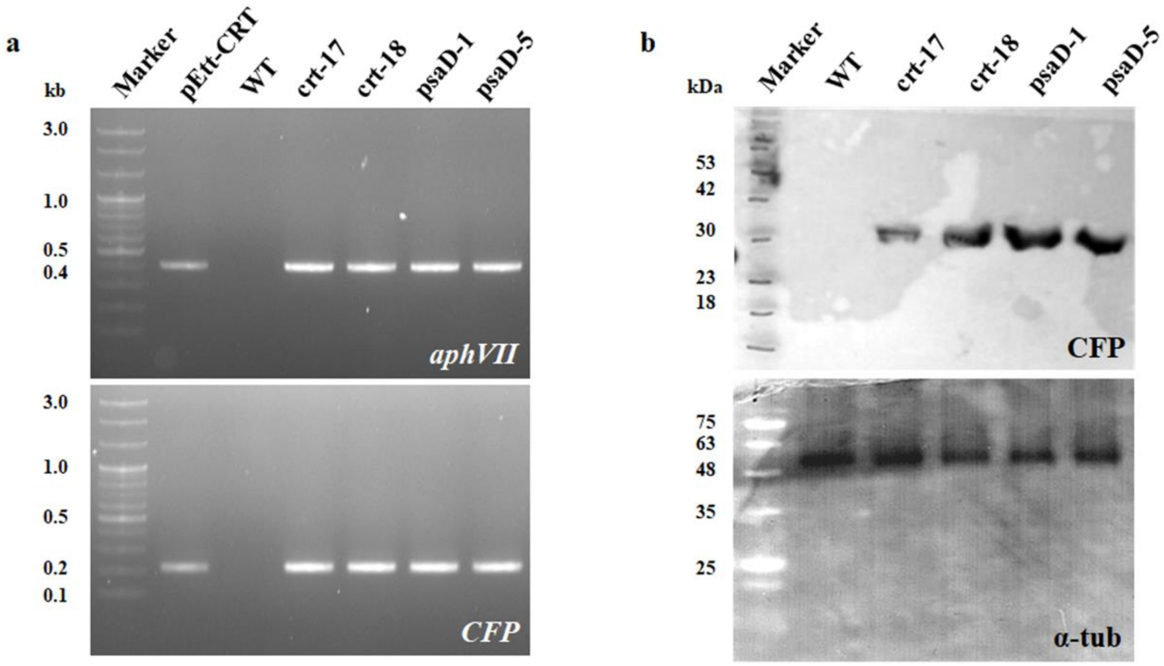
a
Marker
pEtt-CRT
WT
crt-17
crt-18
psaD-1
psaD-5
kb
3.0
1.0
0.5
0.4
aphVII
3.0
1.0
0.5
0.2
0.1
CFP
b
Marker
WT
crt-17
crt-18
psaD-1
psaD-5
kDa
53
42
30
23
18
CFP
75
63
48
35
25
α-tub

Figure S6.

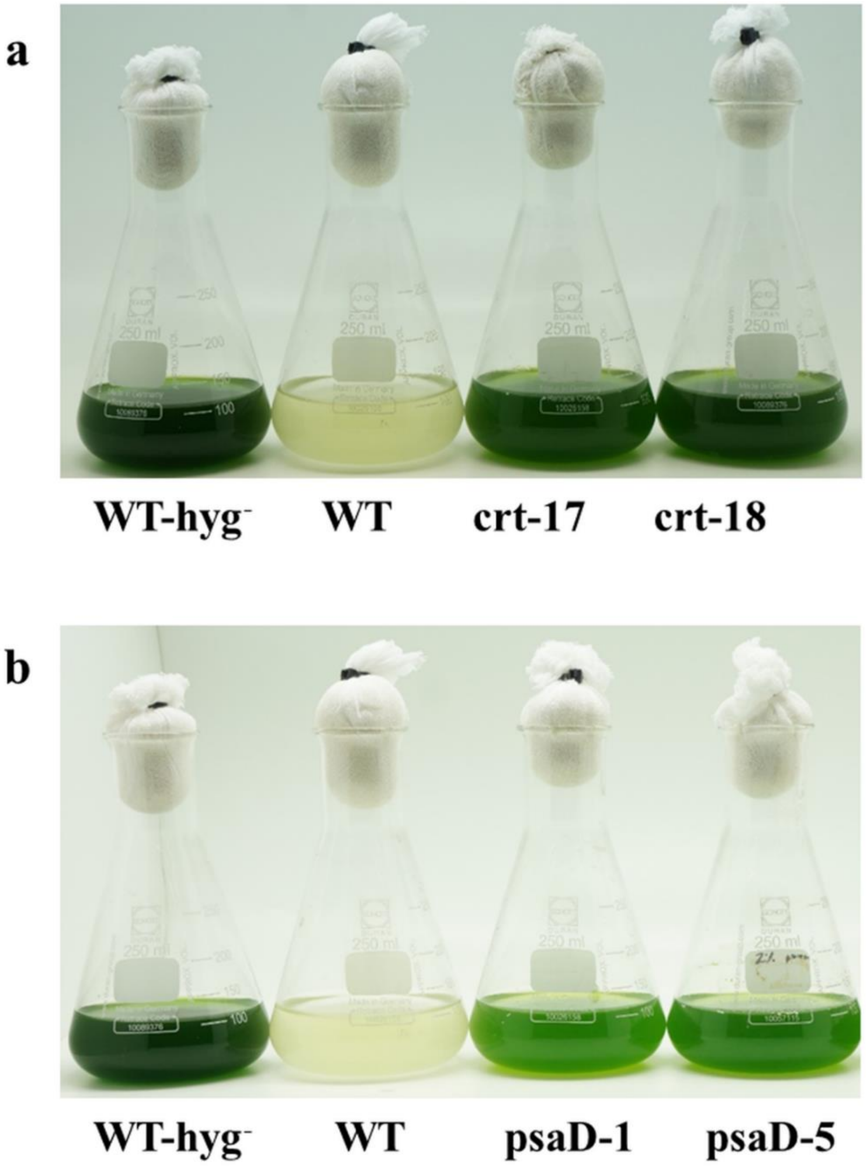

Figure S7.

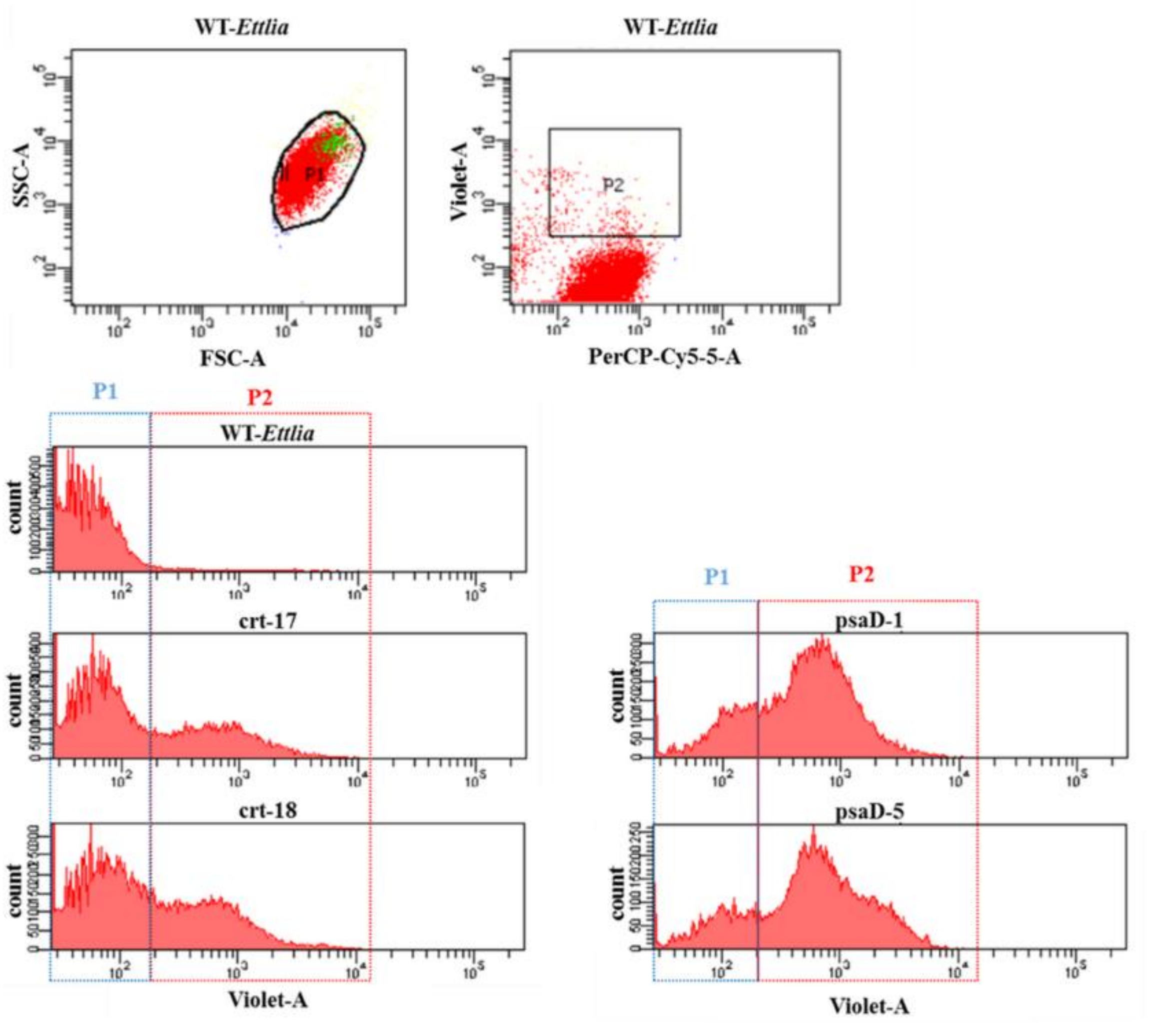
WT-Ettlia
SSC-A
FSC-A
P1
WT-Ettlia
Violet-A
PerCP-Cy5-5-A
P2
P1
P2
WT-Ettlia
crt-17
crt-18
count
Violet-A
P1
P2
psaD-1
psaD-5
count
Violet-A

Figure S8.

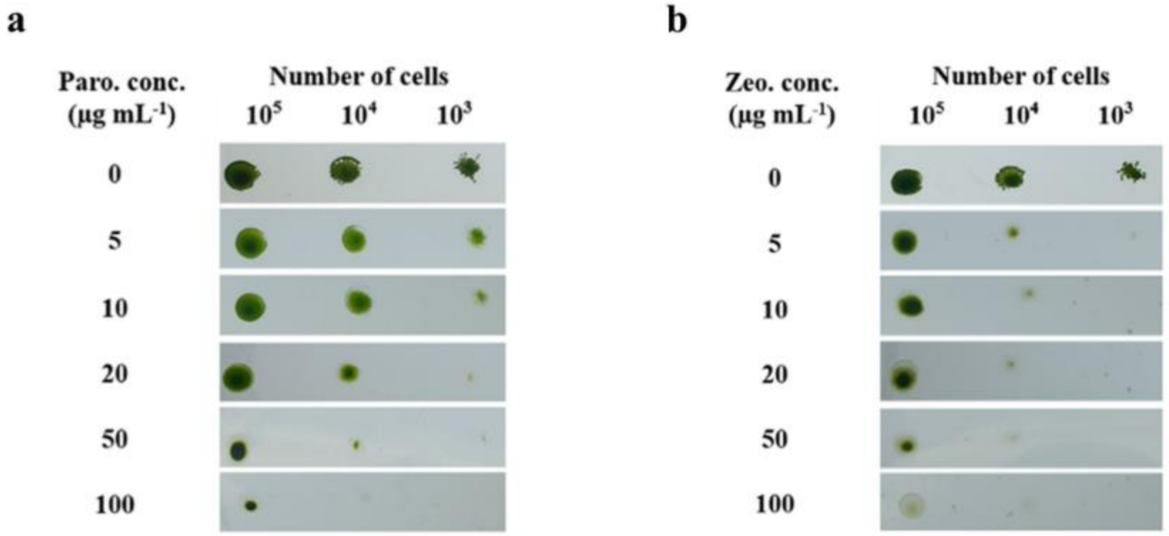
a
Paro. conc.
(μg mL-1)
Number of cells
10^5
10^4
10^3
0
5
10
20
50
100
b
Zeo. conc.
(μg mL-1)
Number of cells
10^5
10^4
10^3
0
5
10
20
50
100

Figure S9.

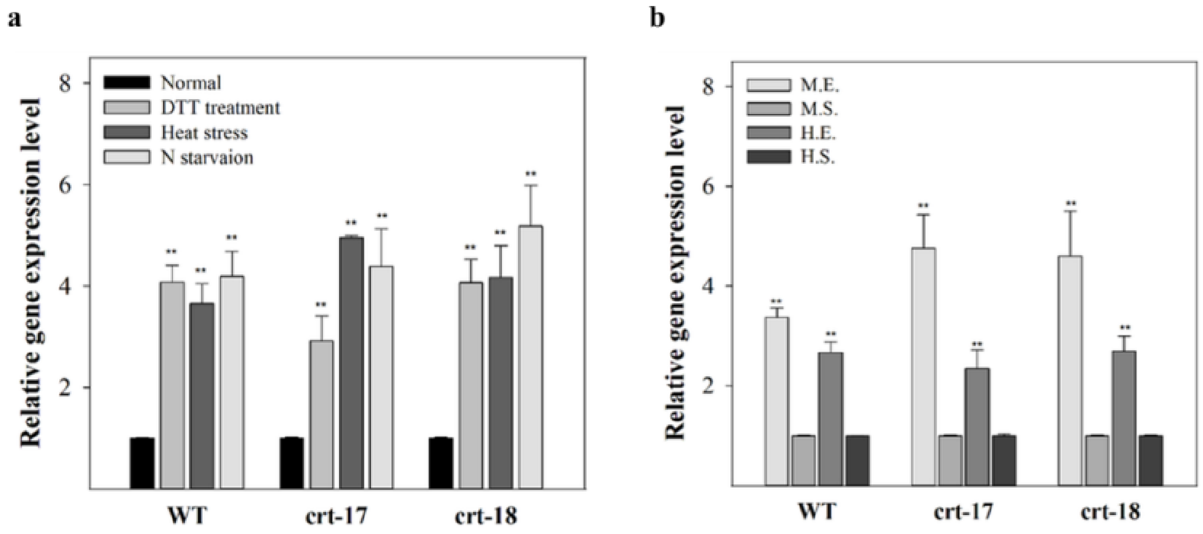
a
Normal
DTT treatment
Heat stress
N starvaion
Relative gene expression level
8
6
4
2
WT
crt-17
crt-18
b
M.E.
M.S.
H.E.
H.S.
Relative gene expression level
8
6
4
2
WT
crt-17
crt-18

Reference

1 Hall, T. A., BioEdit: a user-friendly biological sequence alignment editor and analysis program for Windows 95/98/NT. *Nucleic Acids Symp. Ser.* **41**, 95–98 (1999).
